# Normative data for the BODY-Q module in an Australian population and comparison with North American and European norms

**DOI:** 10.1016/j.jpra.2026.07.011

**Published:** 2026-07-09

**Authors:** Gabrielle E. Shea, Laura Sourdin, Phillipa van Essen, Tamara Crittenden, Nicola R. Dean

**Affiliations:** a College of Medicine and Public Health, Flinders University, Bedford Park, Adelaide, South Australia, Australia; b Department of Plastic and Reconstructive Surgery, Flinders Medical Centre, Bedford Park, Adelaide, South Australia, Australia

**Keywords:** Patient-reported outcome measures, PROM, Quality of life, BODY-Q, Body image

## Abstract

**Background:**

The BODY-Q is a validated patient-reported outcome measure for assessing perceptions of appearance, health-related quality of life and healthcare experiences. Perception of appearance and body image are influenced by cultural, demographic and societal factors that vary across populations. This study aimed to generate Australian normative BODY-Q data. Secondary aims were to understand variation across sociodemographic groups and between geographic regions.

**Methods:**

Participants aged 18 years and older and currently living in Australia provided sociodemographic information and completed appearance and health-related quality of life domains of the BODY-Q. Scale scores were compared across socio-demographic groups, and means compared to published North American and European normative data.

**Results:**

Data were collected from 805 Australian participants. Women reported lower scores than men in four of the five appearance scales and five of the six health-related quality of life scales. Presence of chronic health condition(s) was associated with lower scores in four of the five appearance scales and five of the six health-related quality of life scales. Higher body mass index was associated with lower scores in all BODY-Q scales. Younger age was associated with worse Appearance-related Psychosocial Distress but minimal difference in other scales. Australian participants showed similar scores to Europeans, and both were higher than in North America.

**Conclusion:**

This study provides the first Australian normative scores for the BODY-Q and represents the second publication of normative BODY-Q data internationally. The findings highlight the importance of region-specific normative scores and collecting relevant socio-demographics in patient-reported outcome measures research.

## Introduction

Plastic and reconstructive procedures can influence both a person’s appearance and their health-related quality of life (HRQL). Assessing changes in these domains before and after surgical interventions is therefore helpful for evaluating treatment outcomes. Patient-reported outcome measures (PROMs) are a key component of healthcare, offering insights into individuals’ perspectives on their symptoms, functional status and overall quality of life.^1^^)^ They are particularly valuable within plastic surgery, where patient perceptions often guide both clinical decision-making and assessments of procedural success^1^

The BODY-Q (https://qportfolio.org/body-q) is a validated PROM designed to measure outcomes related to body image and HRQL, particularly in individuals undergoing body contouring, cosmetic and reconstructive surgery.^2^, ^3^, ^4^, ^5^ Despite its increasing use both in Australia and internationally, normative BODY-Q data remains limited. North American and European normative BODY-Q data have been reported by Dalaei et al. in 2022, however, no Australian normative data exists.^6^ Crittenden et al. (2022) demonstrated that normative data for the BREAST-Q differed between regions.^7^ Australian women reported significantly lower BREAST-Q scores than women in the US in four of the five scales; satisfaction with breasts, psychosocial well-being, physical well-being chest and sexual well-being. Similarly, the BODY-Q normative datasets published by Dalaei et al. revealed variability between population groups in North America and Europe.^6^^,^^8^ These findings demonstrate the importance of generating region-specific normative data to ensure accurate, culturally relevant interpretation of PROM scores.

Body image and HRQL are shaped by a range of socio-demographic variables. Previous literature suggests younger individuals tend to experience greater body image concerns,^9^ and women typically report higher levels of body dissatisfaction than men.^10^ In the normative BODY-Q dataset by Dalaei et al., women and younger individuals had lower satisfaction with their appearance and lower HRQL.^6^ Higher Body Mass Index (BMI) and chronic health conditions were also associated with poorer scores.^6^ Understanding how these factors relate to BODY-Q scores within the Australian context is essential for identifying groups that may be at greater risk of reduced body image satisfaction or HRQL.

The objective of this study was to generate normative BODY-Q data from adults in the Australian general population, to examine variation across various socio-demographic groups, and to compare Australian scores with existing normative data from North America and Europe.

## Methods

### Normative population

We used the market research agency, Pureprofile Pty Ltd (Sydney, Australia) to recruit a sample of 805 adults from the Australian general population. Consenting participants aged 18 years and older completed an online survey that included socio-demographic information (age, gender, BMI, presence of chronic health conditions, employment status, education level, and marital status) followed by the BODY-Q appearance and HRQL domains. Participants were reimbursed according to Pureprofile standards. Ethics approval was obtained from the Flinders University Ethics Committee (Approval number 7945–3).

### The BODY-Q

The BODY-Q is a PROM with 30 independently functioning scales that measure 4 domains: Appearance, HRQL, Eating-Related Concerns and Experience of Care. This study used 11 scales from the Appearance and HRQL domains to generate normative data. Appearance scales included: Satisfaction with Body, Satisfaction with Abdomen, Satisfaction with Breasts, Appraisal of Excess Skin, and Appraisal of Stretch Marks. HRQL scales included: Appearance-related Psychosocial Distress, Body Image, Social Function, Psychological Function, Physical Function and Physical Symptoms. Short descriptions of each scale are provided in Supplementary File 1. Each BODY-Q scale has between five and eleven items. Response options are scored between one and four, with higher scores indicating better HRQL / appearance. The only exception is the appearance-related psychosocial distress scale, where higher scores (more distress) indicate worse HRQL.^11^ Raw scores were transformed using Rasch-conversion tables to produce linear interval scores ranging between 0 and 100.^11^ The Rasch transformation allows for more accurate and parametric statistical analysis of PROM scores.^11^

Australian normative BODY-Q data were compared to published North America and European normative BODY-Q data published by Dalaei et al., 2022.^6^ The countries included were Belgium, Canada, Denmark, England, Finland, France, Germany, Italy, Netherlands, Poland, Sweden and United States. Socio-demographic data from the Australian sample was also compared to those of the international samples (Supplementary File 2).

### Statistical analysis

Statistical analyses and figure generation were performed using Microsoft Excel and GraphPad Prism Version 10.0. Descriptive statistics (mean, SD, SEM and 95% confidence intervals) were calculated for each BODY-Q scale incorporated into the study. Socio-demographic variables were compared to 2021 Australian Bureau of Statistics (ABS) Census data using *χ*^2^ test for categorical variables with associated standardised residuals to ensure accurate representation of the Australian population.^12^ BMI for each participant was calculated from self-reported height and weight and organised into categories as per the World Health Organisation; underweight (<18.5 kg/m^2^), normal weight (18.6 - 24.9 kg/m^2^), overweight (25–29.9 kg/m^2^), class 1 obesity (30–34.9 kg/m^2^), class 2 obesity (35–39.9) and class 3 obesity (40 kg/m^2^or greater).^13^ BMI was compared to the 2022 ABS National Health Survey using *χ*^2^ test and Pearson’s correlation analysis.^14^ Age was organised into categories of 18–39 years and ≥ 40 years. One-way Analysis of Variance (ANOVA) was used to assess significance of difference between socio-demographics in the Australian study cohort and the published North American and European cohorts. Two-way ANOVAs were used to assess significance of difference in each BODY-Q scale between various demographic groups. Mean BODY-Q scores were compared with published North American and European scores using two-way ANOVAs. Tukey’s post-hoc method was used for multiple comparisons. Statistical significance was established at *p* < .05.

## Results

### Australian normative study

Normative BODY-Q scores were collected from 805 participants. The mean age was 48 years and ranged from 18 to 89. The cohort included 51% women and 49% men, with the remainder identifying as non-binary/third gender or not stated. Demographic characteristics are reported in Table 1. No significant differences were found for gender or age between the normative study population and the Australian Census data, although there were slightly more participants than expected who were aged ≥40 years in the study cohort. The majority of participants in the study cohort reported working full-time (49%), had a bachelor’s degree level of education (32%), and were married (48%). When compared to the ABS survey data, BMI in the study cohort was found to be statistically different (*p <.001*). The study cohort significantly overrepresented those classified as Underweight (z = 3.08) and Normal Weight (z = 3.12) and significantly underrepresented those with Class 1 obesity (z = −2.31) and Class 2 obesity (z = −4.25). There was no significant difference found for the Overweight or Class 3 obesity categories. Participants in the study cohort reported significantly fewer chronic health conditions when compared to the Australian population (*p <.01*) (Table 1).

**Table 1:** Socio-demographic data of the 805 Australian normative study participants compared with Australian general population reference values.

Variable	Study cohort value	Australian population reference value*	Statistical comparison
Gender			χ²(1) = .004, *p* = .95
Female	410 (50.9%)	51.2%	
Male	392 (48.7%)	48.8%	
Prefer not to say	2 (0.25%)	NA	
Non-binary/third gender	1 (0.12%)	NA	
Age, years			χ²(1) = 2.55, *p* = .11
Mean (SD)	48 (16.7)	38.4	
Median (Range)	47 (18–89)	38 (18–111)	
**18 – 39**	286 (35.5%)	38.3%	
**≥ 40**	519 (64.5%)	61.7%	
BMI, kg/m^2^			χ²(5) = 42.65, p = <.001
**Underweight (≤ 18.5)**	24 (3.0%)	1.6%	*3.08*
**N**ormal weight (**18.6 – 24.9)**	301 (37.4%)	31.6%	*3.12*
Overweight (**25–29.9)**	273 (34.0%)	34.0%	*0.12*
Class 1 obesity (**30–34.9)**	123 (15.3%)	19.0%	*−2.31*
Class 2 obesity (**35–39.9)**	30 (3.7%)	8%	*−4.25*
Class 3 obesity (≥ 40)	37 (4.6%)	4.6%	*.002*
Unable to calculate	17 (2.1%)	NA	
Chronic Health Conditions			χ²(1) = 10.17, *p* = .001
Yes	232 (28.8%)	35.6%	*−2.6*
No	539 (67.0%)	64.4%	*1.9*
Unsure	30 (3.70%)	NA	
Prefer not to say	4 (0.5%)	NA	
Employment Status**			
Working Full-Time	393 (48.8%)	42.8%	
Working Part-Time	153 (19.0%)	13.8%	
Unemployed	21 (2.6%)	3.5%	
Homemaker/Stay at Home Parent	35 (4.3%)	NA	
Student	16 (2.0%)	NA	
Retired	158 (19.6%)	NA	
Voluntary Work	3 (0.4%)	NA	
Unable to Work	21 (2.6%)	NA	
Other	5 (0.6%)	NA	
Education Level**			
Year 11 or Below	79 (9.8%)	NA	
Year 12	148 (18.4%)	NA	
Bachelor Degree	256 (31.8%)	27.5%	
Postgraduate Degree	131 (16.3%)	10.3%	
Trade Certificate or Diploma	188 (23.4%)	33.9%	
Other	3 (0.4%)	NA	
Marital Status			
Married	383 (47.6%)	46.5%	
Living With Partner	121 (15.0%)	NA	
Widowed	21 (2.6%)	5%	
Divorced/Separated	81 (10.1%)	12.1%	
Never Been Married	199 (24.7%)	NA	

*Source for Australian reference values for BMI were taken from the Australian Bureau of Statistics (ABS) National Health Survey in 2022 ^14^. The remainder of the Australian demographic data (age, gender, chronic health conditions, employment status, education level, marital status) was taken from the 2021 ABS Census Data (2021)^12^.

**Employment status and education categories for the study differed to ABS census data therefore not all groups were comparable to the ABS.

Right column for statistical comparison includes *χ*2 (degrees of freedom) and p-value for each category with associated standardised residuals in italics when *χ*2 is significant. Greater than 1.96 indicates significant over-representation and less than −1.96 indicates significant under-representation.

Mean normative BODY-Q scores for each scale within both domains, HRQL and Appearance, are presented in Table 2.

**Table 2:** Mean normative BODY-Q scores from 805 Australian participants.

SCALE	N	MEAN (SD)	95% CI
Satisfaction With Abdomen	802	43.8 (24.5)	42.1–45.5
Satisfaction With Body	803	48.1 (20.3)	46.7–49.6
Appraisal Of Stretch Marks	803	79.2 (25.9)	77.4–81.0
Appraisal Of Excess Skin	803	73.8 (29.1)	71.8–75.9
Satisfaction With Chest	805	55.0 (20.5)	53.5–56.4
Appearance Distress†	805	42.4 (24.1)	40.8–44.1
Body Image	801	48.4 (22.3)	46.9–50.0
Physical Function	802	74.7 (23.9)	73.0–76.3
Psychological Function	802	60.7 (21.9)	59.2–62.2
Sexual Function	796	54.1 (21.6)	52.6–55.6
Social Function	805	56.8 (18.8)	55.5–58.1

† Note appearance distress scale inversion where higher scores indicate worse HRQL (greater appearance-related psychosocial distress).

### Comparison of normative BODY-Q scores between Australian, North American and European cohorts

Comparison of BODY-Q scores across the three geographical regions is shown in Table 3 and demonstrates significant differences across countries. Australians (AUS) reported significantly higher mean scores than both North America (NA) and Europe (EU) in Satisfaction with Abdomen (AUS=43.8 > NA=37, EU=38.4), Appraisal of Excess Skin (AUS=73.8 > NA=48.2, EU=55.1) and Psychological Function (AUS=60.7 > NA=58, EU=57.9). Australia and Europe both had significantly higher scores than North America in Appraisal of Stretch Marks (AUS=79.2, EU=79.7 > NA=71.6), Satisfaction with Chest (AUS=55, EU=55.5 > NA=51.1) and Body Image (AUS=48.4, EU=47.7 > NA=45.3). Australia had significantly lower scores than both North America and Europe in Sexual Function (AUS=54.1 < NA=58.6, EU=61.2). Europe reported the lowest level of Psychosocial Distress compared to Australia (38.8 and 42.4 respectively, *p = .002*). Australian and North American distress scores were not significantly different from each other, likewise no significant differences were found between the Australian cohort for Satisfaction of Body (Europe and North America), Body Image (Europe) or Social Function (Europe).

**Table 3:** Comparison of normative BODY-Q data between Australia and North America and Europe.

	Australia			North America^6^			Difference in means (95% CI)	p-value	Europe^6^			Difference in means (95% CI)	p-value
	n	Mean	SEM	n	Mean	SEM			n	Mean	SEM		
Satisfaction - Abdomen	802	43.8	0.87	1487	37.0	0.71	6.82 (4.4 to 9.2)	<.001	1604	38.4	0.67	5.41 (3.0 to 7.8)	<.001
Satisfaction - Body	803	48.1	0.72	1487	45.8	0.51	2.37 (−0.1 to 4.8)	.06	1993	46.9	0.42	1.27 (−1.0 to 3.6)	.40
Appraisal - Stretchmarks	803	79.2	0.91	857	71.6	0.96	7.53 (4.8 to 10)	<.001	640	79.7	1.15	−0.53 (−3.5 to 2.4)	.91
Appraisal – Excess Skin	803	73.8	1.03	365	48.2	1.50	25.6 (22 to 29)	<.001	222	55.1	2.02	18.8 (14.6 to 23)	<.001
Satisfaction - Chest	805	55.0	0.72	579	51.1	1.06	3.88 (0.9 to 6.9)	.007	792	55.5	0.87	−0.56 (−3.3 to 2.2)	.007
Psychological Distress†	805	42.4	0.85	1487	44.2	0.57	−1.8 (−4.2 to 0.6)	.19	1211	38.8	0.65	3.65 (1.1 to 6.2)	.002
Body Image	801	48.4	0.79	1485	45.3	0.63	3.1 (0.67 to 5.5)	.007	1605	47.7	0.60	0.76 (−1.6 to 3.2)	.74
Physical Function	802	74.7	0.84	1485	81.4	0.56	−6.7 (−9.1 to −4.3)	<.001	1992	80.6	0.56	−5.9 (−8.3 to −3.6)	<.001
Psychological Function	802	60.7	0.77	1485	58.0	0.63	2.66 (0.24 to 5.1)	.027	1605	57.9	0.60	2.8 (0.4 to 5.2)	.017
Sexual Function	796	54.1	0.77	1099	58.6	0.74	−4.5 (−7 to −1.9)	<.001	1277	61.2	0.68	−7.1 (−9.6 to - 4.6)	<.001
Social Function	805	56.8	0.66	1485	54.2	0.54	2.62 (0.2 to 5.0)	.030	1993	55.9	0.45	0.89 (−1.4 to 3.2)	.64

† Note appearance distress scale inversion where higher scores indicate worse HRQL (greater appearance-related psychosocial distress).

### Comparison of Australian BODY-Q scores between participant demographics

**Fig. 1:**
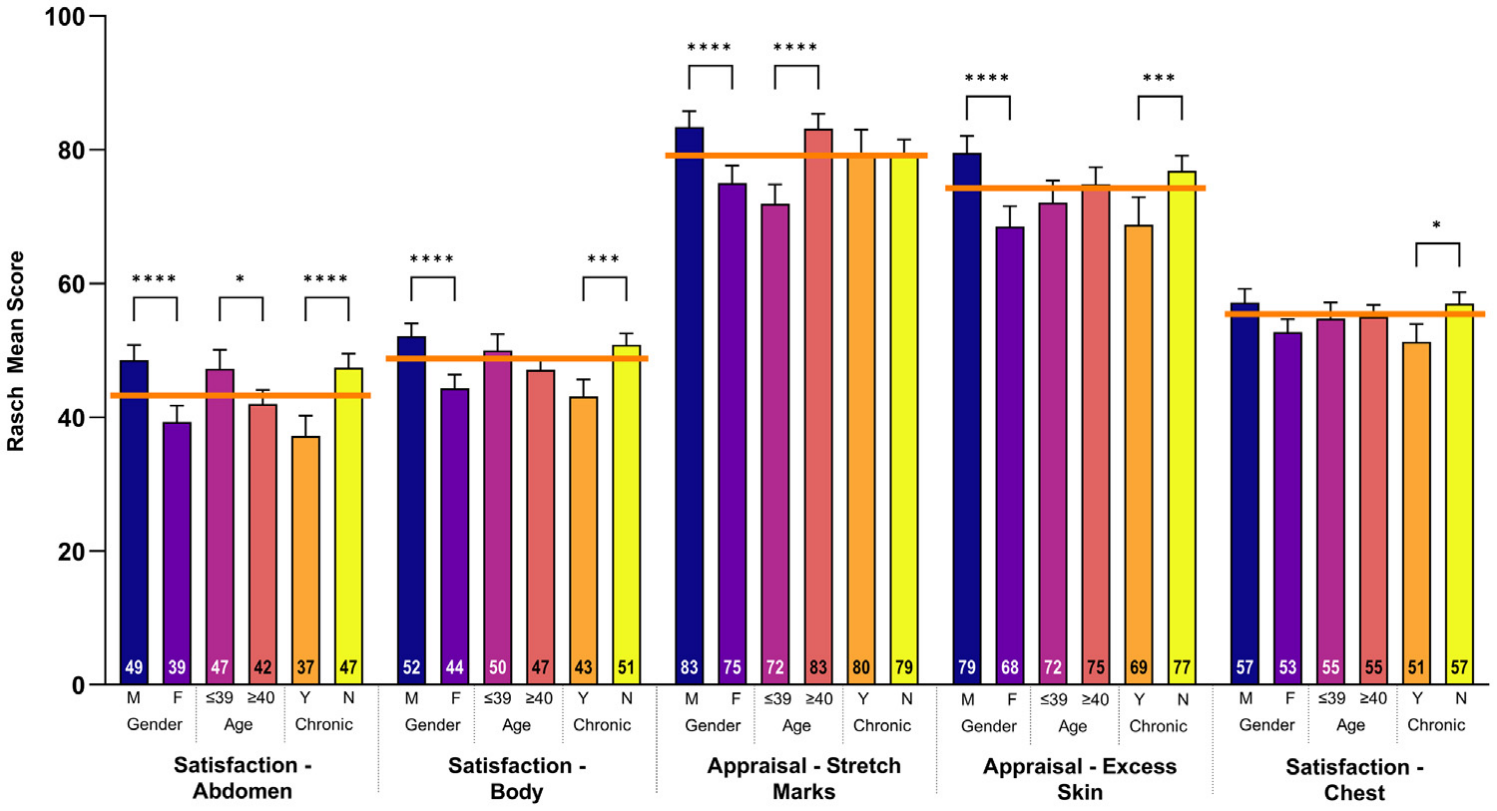
Australian BODY-Q Appearance scales stratified by gender, age and chronic health condition. Each column indicates the mean Rasch score for each group, with the value indicated by the numeral at the base of the column. Error bars indicate 95% confidence interval. Statistical significance was calculated with a Two-way ANOVA between groups indicated by stars, where * = <.05, ** = <.01, *** = <.001, **** = <.0001. Orange lines indicate total mean score for each domain (from Table 2). M= Male, F= Female, Y= Yes, N= No.

**Fig. 2:**
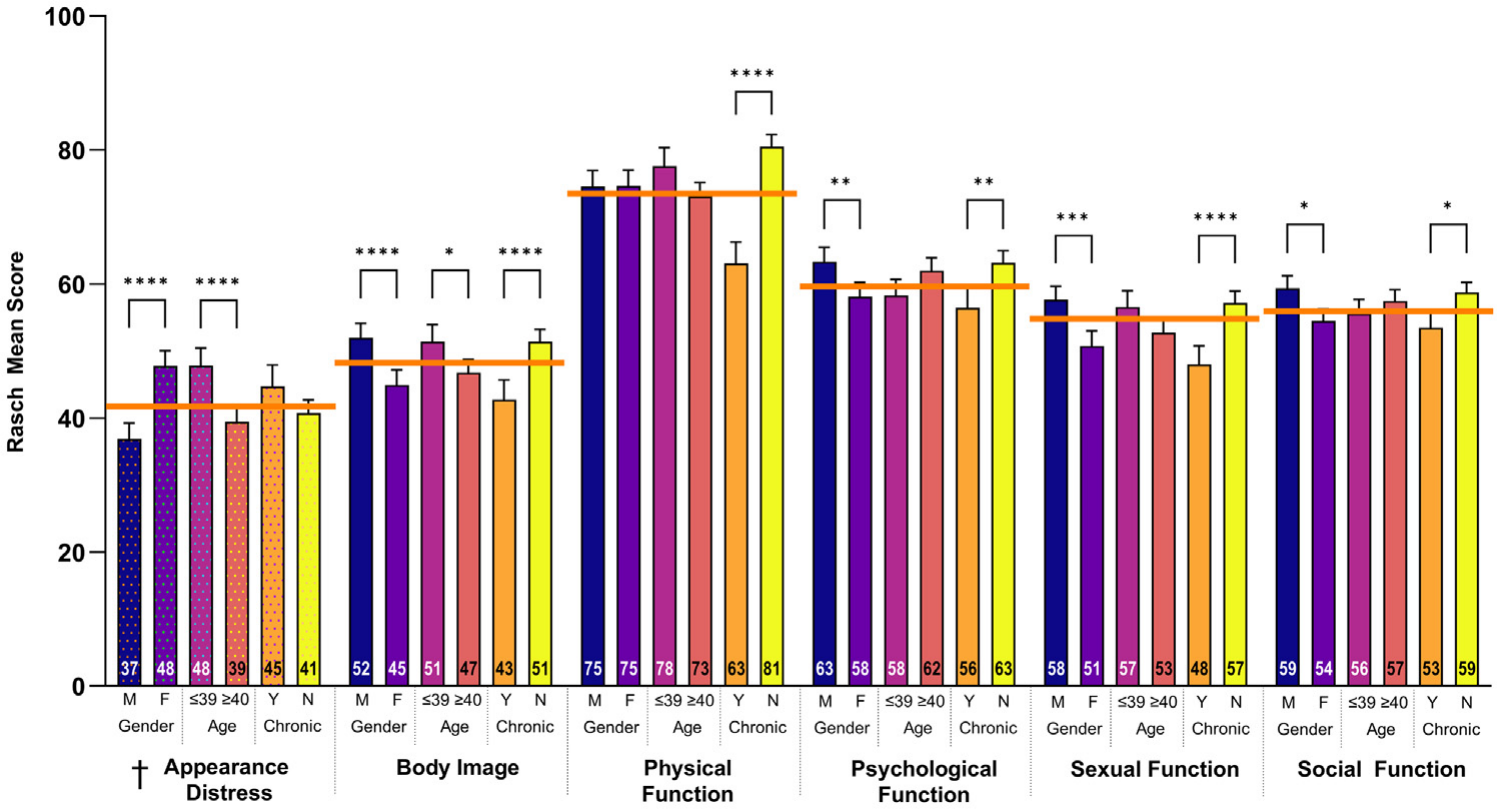
Australian BODY-Q Health-Related Quality of Life (HRQL) scales stratified by gender, age and chronic health condition. Each column indicates the mean Rasch score for each group, with the value indicated by the numeral at the base of the column. Error bars indicate 95% confidence interval. Statistical significance was calculated with a Two-way ANOVA between groups indicated by stars, where * = <.05, ** = <.01, *** = <.001, **** = <.0001. Orange lines indicate total mean score for each domain (Table 2). M= Male, F= Female, Y= Yes, N= No. ^†^ Note appearance distress scale inversion where higher scores indicate worse HRQL (greater appearance-related psychosocial distress).

**Fig. 3:**
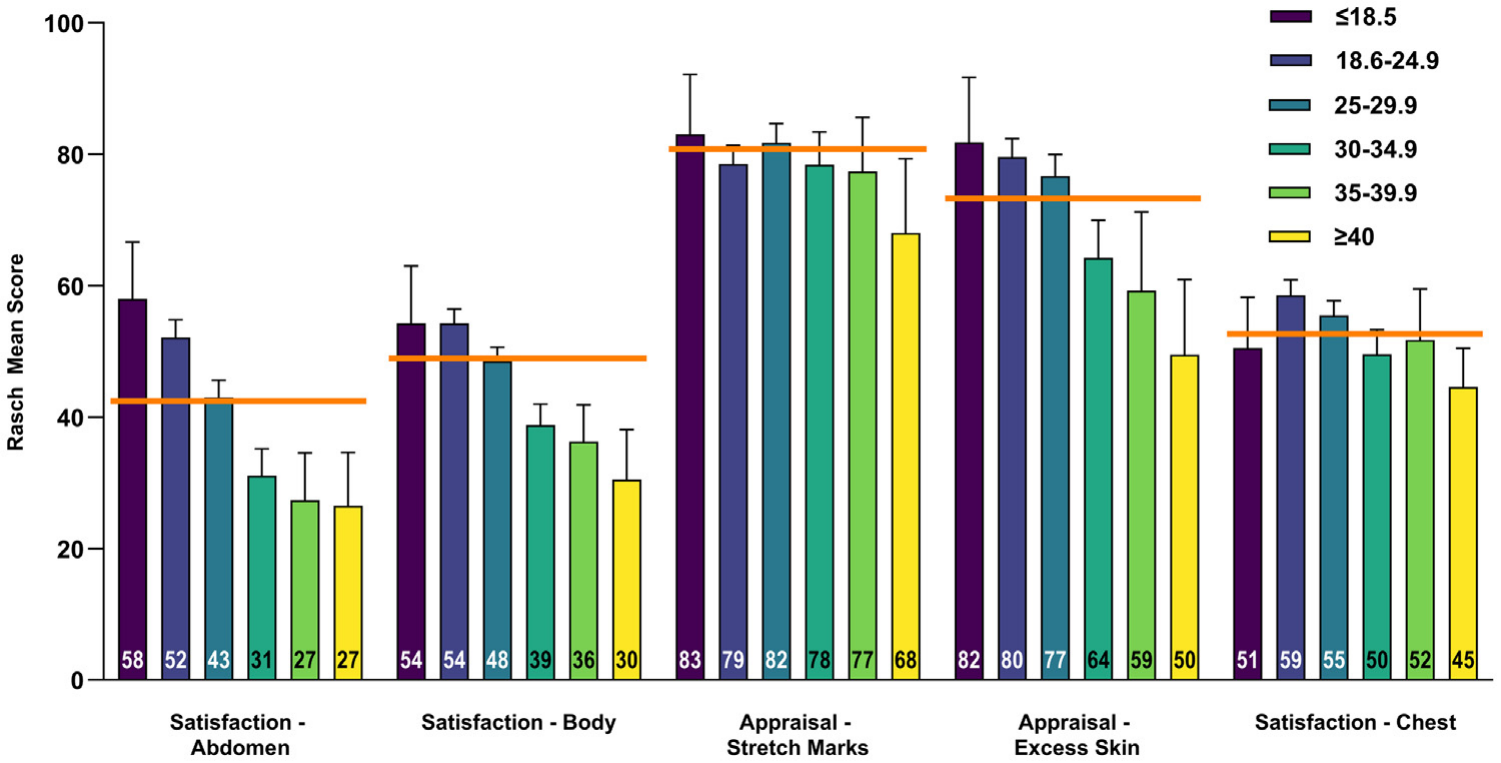
Australian BODY-Q Appearance scales stratified by body mass index (BMI). Each column indicates the mean Rasch score for each group, with the value indicated by the numeral at the base of the column. Error bars indicate 95% confidence interval. BMI groups indicated in legend (kg/m^2^). Orange lines indicate total mean score for each domain (from Table 2).

**Fig. 4:**
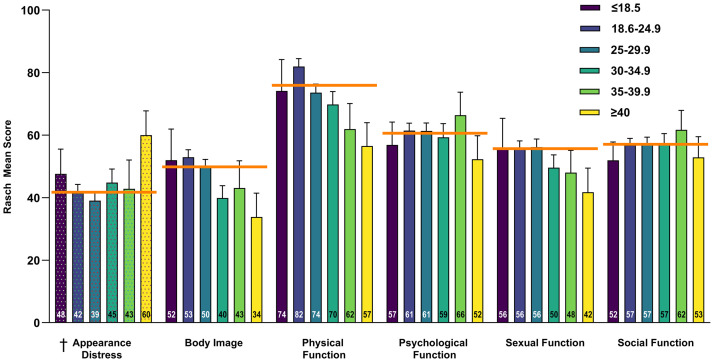
Australian BODY-Q Health-Related Quality of Life (HRQL) scales stratified by body mass index (BMI). Each column indicates the mean Rasch score for each group, with the value indicated by the numeral at the base of the column. Error bars indicate 95% confidence interval. BMI groups indicated in legend (kg/m^2^). Orange lines indicate total mean score for each domain (from Table 2). ^†^ Note appearance distress scale inversion where higher scores indicate worse HRQL (greater appearance-related psychosocial distress).

Mean normative BODY-Q scores compared across various demographic groups are shown in Fig. 1, Fig. 2, Fig. 3, Fig. 4. Fig. 1 and Fig. 2 display the BODY-Q Appearance and HRQL domains respectively across three variables: gender (men vs women), age (≤39 vs ≥ 40 years) and presence of one or more chronic health conditions (yes vs no). Female participants had significantly lower scores than male participants across all scales except for Satisfaction with Chest and Physical Function (*p = <.05*). Women had significantly worse scores than men for the Appearance-related Psychosocial Distress scale, indicating greater distress. Age had minimal impact on appearance and HRQL scales, except for Appearance-related Psychosocial Distress where participants aged 39 years and younger had significantly worse levels of distress than those 40 years and older.

Participants reporting one or more chronic health conditions had significantly lower scores in four of the five Appearance scales (p = <.05)(see Fig. 1) and five of the six HRQL scales (*p = <.05*)(see Fig. 2).

Normative BODY-Q scores across BMI categories are shown in Fig. 3 (Appearance) and Fig. 4 (HRQL). For most scales, BODY-Q scores were shown to decrease as BMI increased. Pearson correlation analysis showed that higher BMI was significantly associated with lower scores for Satisfaction with Abdomen, Satisfaction with Body, Satisfaction with Chest, Body Image, Physical Function, and Sexual Function (*p < .0001*). Appearance-related Psychosocial Distress demonstrated a weak positive correlation with BMI (r =.12, *p =.001*), noting the inverse score. No significant associations were observed between BMI and Appraisal of Stretch Marks, Psychological Function, and Social Function (Table 4).

**Table 4:** Pearson correlation analysis of BMI groups vs BODY-Q scales.

BODY-Q Scale	r	r^2^	N	t	p-value
Satisfaction With Abdomen	-.35	.12	773	−10.4	< .001
Satisfaction With Body	-.33	.11	773	−9.6	< .001
Appraisal Of Stretch Marks	-.05	.003	773	−1.5	.13
Appraisal Of Excess Skin	-.25	.06	773	−7.5	< .001
Satisfaction With Chest	-.16	.03	773	−4.6	< .001
Appearance Distress†	.12	.01	773	3.3	.001
Body Image	-.24	.06	773	−6.9	< .001
Physical Function	-.26	.07	773	−7.5	< .001
Psychological Function	-.07	.005	773	−1.5	.13
Sexual Function	-.16	.03	773	−4.5	< .001
Social Function	-.01	.001	773	−0.3	.77

† Note appearance distress scale inversion where higher scores indicate worse HRQL (greater appearance-related psychosocial distress).

### Comparison of socio-demographic variables between Australian, North American and European normative cohorts

Socio-demographic comparisons between the Australian and the North American and European normative cohorts from Dalaei et al.^6^ are detailed in Supplementary File 2. Significant differences were observed across most variables including age, gender, employment status, and marital status. The North American cohort had significantly more women, and the European had significantly more men, than the Australian cohort. The Australian normative cohort was significantly older than both the North American and European cohorts (AUS=48yrs > NA=43yrs, EU=29yrs, *p < .001*). BMI distributions differed significantly between countries, where the mean of the Australian cohort was 27 kg/m^2^, compared to 28.14 kg/m^2^ in North America (mean difference to Australia of −1.14 [95% CI, −1.77 to −0.51], *p <.0001*) and 24.55 kg/m^2^ in Europe (mean difference to Australia of 2.45 [95% CI, 1.82 to 3.10], *p <.0001*).

## Discussion

Measurements of appearance and quality of life are key outcomes in obesity and body contouring research and require standardised, validated PROMs.^15^ The development and utilisation of PROMs is not only helpful for informed research but also for clinical practice, particularly within plastic and reconstructive surgery. The BODY-Q is a validated PROM which is widely utilised worldwide, particularly for reporting outcomes following weight loss or body contouring surgery from the patient perspective.^2^^,^^4^^,^^6^ Currently, the only available normative scores for the BODY-Q come from the North American and European populations.^6^ Prior research has demonstrated meaningful differences between PROM normative values in Australia compared to other countries.^7^ This is the first study to produce Australian normative BODY-Q scores, enabling contextually appropriate interpretation of BODY-Q scores in both research and clinical practice.

A total of eleven BODY-Q scales were evaluated across two domains: Appearance and HRQL. Sociodemographic factors are well established determinants of body image and health-related quality of life. Previous research has demonstrated that age, gender, BMI and chronic health conditions significantly influence both appearance-related distress and broader HRQL outcomes across a range of PROMs, including the EQ-5D and other generic and condition-specific measures.^6^^,^^9^^,^^10^^,^^16^ Understanding the influence of these variables is therefore important when establishing normative reference values. The BODY-Q has enabled a much more sophisticated understanding of different elements of appearance and HRQL and this study aids in addressing how socio-demographics can influence these different elements.

Across the Australian cohort, younger participants (39 and younger) reported significantly greater distress about their appearance than older people, aligning with prior literature indicating that younger individuals experience increased body image concerns.^9^ The minimal variation between age groups in other scales contrasts with findings from North American and European cohorts where younger age was negatively associated with all appearance and HRQL scales^6^^,^^17^ Conversely, another European study has demonstrated higher normative scores for younger age, when using EQ-5D-3 L, a similar generic measure of health.^18^ These results may indicate variability within different continents or alternatively different sensitivities with alternative PROMs. Normative BREAST-Q scores of the Australian population indicate variation across specific scales, with those aged 40 years and above having lower distress about their appearance, which is similar to the Australian BODY-Q study, highlighting the need for Australian specific data in PROM research.^7^

Gender-related disparities similarly reflect well-established sociodemographic influences on body image. The pattern of women having consistently lower scores than men aligns with extensive literature demonstrating poorer body image in women compared to men and highlights the importance of considering the use of gender-specific normative values in both clinical interpretation and development of future PROMs.^6^^,^^10^^,^^18^, ^19^, ^20^ A similar pattern was evident among individuals reporting chronic health conditions, which parallels other studies demonstrating adverse effects of chronic health on health-related quality of life and body dissatisfaction using other PROMs.^21^^,^^22^

BMI emerged as a particularly influential determinant across both Appearance and HRQL domains, which is consistent with North American and European normative data.^6^ These findings reflect multiple other generic PROMS including SF-36, IWQOL-Lite, and BREAST-Q.^7^^,^^23^^,^^24^ These findings emphasise the significance of obesity as a chronic health condition and the impact on health-related quality of life and body image.

Overall, North America tended to have lower scores than Australia and Europe, which had similar scores to each other for many of the scales reported. Existing studies also demonstrate differences between Australia and other countries in other PROMs including SF-36, SF-6D and EQ-5D^25^, ^26^, ^27^ Interestingly, despite comparable obesity prevalence in Australia and North America, the magnitude of BMI-associated differences in BODY-Q scores appears greater in North American cohorts.^6^ This may reflect sociocultural differences in weight perception, body ideals, or the internalisation of appearance norms across populations.

A key strength of this study is the generation of the first Australian population normative dataset for the BODY-Q, addressing a gap in existing PROM literature. The large sample of 805 participants provides a strong foundation for subgroup analyses, enabling comparisons across gender, age, BMI and chronic health conditions.

One of the limitations of this study was the reporting of BMI. Our cohort was found to over-represent those with a lower BMI and under-represent those with a higher BMI.^14^ This likely reflects the ‘healthy participant effect’, and participation biases associated with voluntary online recruitment.^28^, ^29^, ^30^ It is difficult to determine the impact of this bias in this particular study. The authors acknowledge that the reported differences in BMI between countries may not be as great if the study cohort were more representative of the underlying Australian population. Demographic data between the Australian population and the North American and European population also differed significantly across age, which may partially account for the regional variation observed. However, our study demonstrated that age had minimal impact on the majority of BODY-Q scores within the Australian population.

Recruitment for the study was through Pureprofile, an online crowdsourcing platform, which although widely used in normative PROM research, may not fully capture the gender diversity of the general population. Only two participants identified outside of the male-female binary, restricting analyses across gender-diverse groups. Binary measurement of gender means it is unknown whether transgender participants (including trans men and women) identified within the “male” or “female” categories. This should be improved in future studies with the updated Australian National Health and Medical Research Council (NHMRC) and ABS standards for sex and gender data collection from late 2025.^31^

In conclusion, this study has established the first set of normative data for the BODY-Q in the Australian population. This allows for better contextual understanding of body image and health-related quality of life in the general population, and how socio-demographics can influence this. This dataset can be used in future research to compare PROMs for individual people against an established baseline, before and after intervention. BMI, presence of chronic health condition(s) and female gender were generally associated with lower scores in the majority of Appearance and HRQL scales. Older age was associated with less appearance-related distress. This study demonstrated relative similarities between Australia and Europe, which were overall higher scores than North America. This highlights the importance of region-specific normative scores and collecting relevant participant socio-demographics when conducting PROM studies.

This study also contributes to the broader body of global health knowledge by adding only the second publication of normative BODY-Q data internationally. This enhances the opportunity for comparisons of body image and HRQL outcomes across different regions. Additionally, the findings from this study could inform public health strategies aimed at improving body image and quality of life, particularly for at-risk groups within the Australian population such as women and those with higher BMI. The BODY-Q provides a benchmark against which individual patient scores can be interpreted, supporting pre-operative counselling, shared decision-making and evaluation of treatment outcomes in both surgical and non-surgical settings.

## Financial disclosure statement

No funding was received for conducting this study.

## Statement of ethical approval

Ethics approval for this study was obtained from the Flinders University Ethics Committee (Approval number 7945-3).

## Conflict of interest statement

None.

## Appendix Supplementary materials

Image, application 1

Image, application 2
