# Supplementary material for: Normative data for the BODY-Q module in an Australian population and comparison with North American and European norms
Source: JPRAS Open. 2026 Jul 9;51:402–13. doi: 10.1016/j.jpra.2026.07.011 (PMC13445518; doi:10.1016/j.jpra.2026.07.011)
Supplement: Supplementary file 1 [file mmc1.docx]

**Supplementary File 1:** Shortened items for BODY-Q scales^12^

Included in BODY-Q Australian normative study: Appearance (Abdomen, Body, Chest, Skin, Stretch Marks) and HRQOL (Appearance distress, Body Image, Physical Function, Physical Symptoms, Psychological function, Sexual Function, Social Function).
