# Supplementary material for: Normative data for the BODY-Q module in an Australian population and comparison with North American and European norms
Source: JPRAS Open. 2026 Jul 9;51:402–13. doi: 10.1016/j.jpra.2026.07.011 (PMC13445518; doi:10.1016/j.jpra.2026.07.011)
Supplement: Supplementary file 2 [file mmc2.docx]

**Supplementary File 2:** Participant socio-demographics of the Australian BODY-Q normative study compared to North America and Europe study ^6^

| Variable | AUSTRALIA | North america | euRope |
| --- | --- | --- | --- |
| Gender |  |  |  |
| Female | 410 (50.9%) | 1191 (58.0%) | 805 (40.3%) |
| Male | 392 (48.7%) | 849 (41.4%) | 1174 (58.7%) |
| Other | 3 (0.37%) | 12 (0.6%) | 20 (1.0%) |
| Age, Mean years (SD)) | 48 (16.7) | 43 (14.7) | 29 (9.72%) |
| Difference In Means (95% CI) |  | 5 (3.71-6.30) | 19.2 (13.2-15.2) |
| P-Value |  | <0.0001 | <0.0001 |
| BMI, kg/m^2 ,^ Mean (SD) | 27.0 (6.43) | 28.1 (7.43) | 24.6 (5.19) |
| Difference In Means (95% CI) |  | -1.14 (-1.77 - -0.51) | 2.45 (1.82 – 3.10) |
| P-Value |  | <0.0001 | <0.0001 |
| BMI groups, kg/m^2^ |  |  |  |
| **≤ 18.5** | 24 (3.0%) | 61 (3.0%) | 133 (6.7%) |
| 18.6 – 24.9 | 301 (37.6%) | 766 (37.3%) | 1047 (75.4%) |
| 25-29.9 | 273 (34.1%) | 588 (28.6%) | 451 (22.6%) |
| 30-34.9 | 123 (15.4%) | 328 (16.0%) | 166 (8.3%) |
| 35-39.9 | 30 (3.75%) | 153 (7.5%) | 71 (3.6%) |
| ≥ 40 | 37 (4.62%) | 157 (7.6%) | 30 (1.5%) |
| Employment Status |  |  |  |
| Working Full-Time | 393 (48.8%) | 879 (42.8%) | 662 (33.0%) |
| Working Part-Time | 153 (19.0%) | 291 (14.2%) | 285 (14.2%) |
| Unemployed | 21 (2.6%) | 188 (9.2%) | 253 (12.6%) |
| Homemaker/Stay At Home Parent | 35 (4.3%) | 135 (6.6%) | 60 (3.0%) |
| Student | 16 (2.0%) | 158 (7.7%) | 722 (36.0%) |
| Retired | 158 (19.6%) | 176 (8.6%) | 26 (1.3%) |
| Voluntary Work | 3 (0.4%) | 26 (1.3%) | 24 (1.2%) |
| Unable To Work | 21 (2.6%) | 88 (4.3%) | 38 (1.9%) |
| Other | 5 (0.6%) | 9 (0.4%) | 14 (0.7%) |
| Marital Status |  |  |  |
| Married | 383 (47.6%) | 825 (40.2%) | 313 (15.7%) |
| Living With Partner | 121 (15.0%) | 142 (6.9%) | 406 (20.3%) |
| Widowed | 21 (2.6%) | 44 (2.1%) | 4 (0.2%) |
| Divorced/Separated | 81 (10.1%) | 265 (13%) | 49 (2.5%) |
| Never Been Married | 199 (24.7%) | 776 (37.8%) | 1227 (61.4%) |
